# Supplementary material for: In situ architecture of the human prohibitin complex
Source: Nat Cell Biol. 2025 Mar 21;27(4):633–40. doi: 10.1038/s41556-025-01620-1 (PMC11991916; doi:10.1038/s41556-025-01620-1)
Supplement: Supplementary file 1 — Reporting Summary [file 41556_2025_1620_MOESM1_ESM.pdf]

nature portfolio

Corresponding author(s): Stefan Jakobs
Last updated by author(s): Jan 14, 2025

Reporting Summary

Nature Portfolio wishes to improve the reproducibility of the work that we publish. This form provides structure for consistency and transparency in reporting. For further information on Nature Portfolio policies, see our Editorial Policies and the Editorial Policy Checklist.

Statistics

For all statistical analyses, confirm that the following items are present in the figure legend, table legend, main text, or Methods section.

n/a	Confirmed	
☐	☒	The exact sample size (*n*) for each experimental group/condition, given as a discrete number and unit of measurement
☐	☒	A statement on whether measurements were taken from distinct samples or whether the same sample was measured repeatedly
☐	☒	The statistical test(s) used AND whether they are one- or two-sided<br>*Only common tests should be described solely by name; describe more complex techniques in the Methods section.*
☒	☐	A description of all covariates tested
☐	☒	A description of any assumptions or corrections, such as tests of normality and adjustment for multiple comparisons
☐	☒	A full description of the statistical parameters including central tendency (e.g. means) or other basic estimates (e.g. regression coefficient) AND variation (e.g. standard deviation) or associated estimates of uncertainty (e.g. confidence intervals)
☐	☒	For null hypothesis testing, the test statistic (e.g. F, t, r) with confidence intervals, effect sizes, degrees of freedom and P value noted<br>*Give P values as exact values whenever suitable.*
☒	☐	For Bayesian analysis, information on the choice of priors and Markov chain Monte Carlo settings
☒	☐	For hierarchical and complex designs, identification of the appropriate level for tests and full reporting of outcomes
☒	☐	Estimates of effect sizes (e.g. Cohen's *d*, Pearson's *r*), indicating how they were calculated

Our web collection on statistics for biologists contains articles on many of the points above.

Software and code

Policy information about availability of computer code

Data collection	SerialEM 4.0, Cryo AutoTEM, LAS AF, BD FACSDiva, TVIPS EM-Menu
Data analysis	Relion 4.0, Dynamo 1.1.532, IMOD 4.11, MODELLER 10.4, PyMOL 2.5, GROMACS, GROmaps, IsoNet 0.2, Excel 2016, Blender 4.2

For manuscripts utilizing custom algorithms or software that are central to the research but not yet described in published literature, software must be made available to editors and reviewers. We strongly encourage code deposition in a community repository (e.g. GitHub). See the Nature Portfolio guidelines for submitting code & software for further information.

Data

Policy information about availability of data

All manuscripts must include a data availability statement. This statement should provide the following information, where applicable:

- Accession codes, unique identifiers, or web links for publicly available datasets
- A description of any restrictions on data availability
- For clinical datasets or third party data, please ensure that the statement adheres to our policy

Data will be available under accessions EMD-19459 (EMDB) and PDB ID 8RRH (PDB). The mass spectrometry data used for analysis in this study was cited at multiple occasions for the readers to follow. Source data have been provided in Source Data. All other data supporting the findings of this study are available from the corresponding author on reasonable request.

Research involving human participants, their data, or biological material

Policy information about studies with human participants or human data. See also policy information about sex, gender (identity/presentation), and sexual orientation and race, ethnicity and racism.

Reporting on sex and gender	N/A
Reporting on race, ethnicity, or other socially relevant groupings	N/A
Population characteristics	N/A
Recruitment	N/A
Ethics oversight	N/A

Note that full information on the approval of the study protocol must also be provided in the manuscript.

Field-specific reporting

Please select the one below that is the best fit for your research. If you are not sure, read the appropriate sections before making your selection.

☒ Life sciences ☐ Behavioural & social sciences ☐ Ecological, evolutionary & environmental sciences

For a reference copy of the document with all sections, see nature.com/documents/nr-reporting-summary-flat.pdf

Life sciences study design

All studies must disclose on these points even when the disclosure is negative.

Sample size	All experiments were conducted at least in triplicate using independent sample preparations. Sample size were chosen based on previous experience and standards in the field. For cryoET data sets the maximum available samples were used for data acquisition.
Data exclusions	No data was excluded to ensure a comprehensive representation of our findings.
Replication	All attempts to replication were successful. Samples were replicated in at least three independent wells.
Randomization	In this study, controlling for covariates was not directly feasible due to the structure of the experimental design. Specifically, the samples were systematically arranged on culture plates and cryo EM grids without employing randomization thereby ensuring equal representation of all conditions. This specific organization minimized the impact of potential unforeseen variables resulting in reliable and consistent data for analysis.
Blinding	Blinding was not possible in this study, as one individual conducted most of the light microscopy analysis and another handled the cryo-ET analysis. However, to minimize potential bias additional authors participated in validating the analysis and thereby ensuring the objectivity of the results.

Behavioural & social sciences study design

All studies must disclose on these points even when the disclosure is negative.

Study description	*Briefly describe the study type including whether data are quantitative, qualitative, or mixed-methods (e.g. qualitative cross-sectional, quantitative experimental, mixed-methods case study).*
Research sample	*State the research sample (e.g. Harvard university undergraduates, villagers in rural India) and provide relevant demographic information (e.g. age, sex) and indicate whether the sample is representative. Provide a rationale for the study sample chosen. For studies involving existing datasets, please describe the dataset and source.*
Sampling strategy	*Describe the sampling procedure (e.g. random, snowball, stratified, convenience). Describe the statistical methods that were used to predetermine sample size OR if no sample-size calculation was performed, describe how sample sizes were chosen and provide a rationale for why these sample sizes are sufficient. For qualitative data, please indicate whether data saturation was considered, and what criteria were used to decide that no further sampling was needed.*
Data collection	*Provide details about the data collection procedure, including the instruments or devices used to record the data (e.g. pen and paper, computer, eye tracker, video or audio equipment) whether anyone was present besides the participant(s) and the researcher, and whether the researcher was blind to experimental condition and/or the study hypothesis during data collection.*

Timing	*Indicate the start and stop dates of data collection. If there is a gap between collection periods, state the dates for each sample cohort.*
Data exclusions	*If no data were excluded from the analyses, state so OR if data were excluded, provide the exact number of exclusions and the rationale behind them, indicating whether exclusion criteria were pre-established.*
Non-participation	*State how many participants dropped out/declined participation and the reason(s) given OR provide response rate OR state that no participants dropped out/declined participation.*
Randomization	*If participants were not allocated into experimental groups, state so OR describe how participants were allocated to groups, and if allocation was not random, describe how covariates were controlled.*

Ecological, evolutionary & environmental sciences study design

All studies must disclose on these points even when the disclosure is negative.

Study description	*Briefly describe the study. For quantitative data include treatment factors and interactions, design structure (e.g. factorial, nested, hierarchical), nature and number of experimental units and replicates.*
Research sample	*Describe the research sample (e.g. a group of tagged Passer domesticus, all Stenocereus thurberi within Organ Pipe Cactus National Monument), and provide a rationale for the sample choice. When relevant, describe the organism taxa, source, sex, age range and any manipulations. State what population the sample is meant to represent when applicable. For studies involving existing datasets, describe the data and its source.*
Sampling strategy	*Note the sampling procedure. Describe the statistical methods that were used to predetermine sample size OR if no sample-size calculation was performed, describe how sample sizes were chosen and provide a rationale for why these sample sizes are sufficient.*
Data collection	*Describe the data collection procedure, including who recorded the data and how.*
Timing and spatial scale	*Indicate the start and stop dates of data collection, noting the frequency and periodicity of sampling and providing a rationale for these choices. If there is a gap between collection periods, state the dates for each sample cohort. Specify the spatial scale from which the data are taken*
Data exclusions	*If no data were excluded from the analyses, state so OR if data were excluded, describe the exclusions and the rationale behind them, indicating whether exclusion criteria were pre-established.*
Reproducibility	*Describe the measures taken to verify the reproducibility of experimental findings. For each experiment, note whether any attempts to repeat the experiment failed OR state that all attempts to repeat the experiment were successful.*
Randomization	*Describe how samples/organisms/participants were allocated into groups. If allocation was not random, describe how covariates were controlled. If this is not relevant to your study, explain why.*
Blinding	*Describe the extent of blinding used during data acquisition and analysis. If blinding was not possible, describe why OR explain why blinding was not relevant to your study.*

Did the study involve field work? ☐ Yes ☐ No

Field work, collection and transport

Field conditions	*Describe the study conditions for field work, providing relevant parameters (e.g. temperature, rainfall).*
Location	*State the location of the sampling or experiment, providing relevant parameters (e.g. latitude and longitude, elevation, water depth).*
Access & import/export	*Describe the efforts you have made to access habitats and to collect and import/export your samples in a responsible manner and in compliance with local, national and international laws, noting any permits that were obtained (give the name of the issuing authority, the date of issue, and any identifying information).*
Disturbance	*Describe any disturbance caused by the study and how it was minimized.*

Reporting for specific materials, systems and methods

We require information from authors about some types of materials, experimental systems and methods used in many studies. Here, indicate whether each material, system or method listed is relevant to your study. If you are not sure if a list item applies to your research, read the appropriate section before selecting a response.

Materials & experimental systems

n/a	Involved in the study	
☐	☒	Antibodies
☐	☒	Eukaryotic cell lines
☒	☐	Palaeontology and archaeology
☐	☒	Animals and other organisms
☒	☐	Clinical data
☒	☐	Dual use research of concern
☒	☐	Plants

Methods

n/a	Involved in the study	
☒	☐	ChIP-seq
☐	☒	Flow cytometry
☒	☐	MRI-based neuroimaging

Antibodies

Antibodies used

anti-PHB1 (EP2803Y, 1:2000, Abcam, Cambridge, UK), anti-PHB2 (EPR14523, 1:5000, Abcam, Cambridge, UK), anti-GFP (JL-8, 1:3000, Clontech, Saint-Germain-en-Laye, France), anti-Actin (AC74, 1:3000, Sigma-Aldrich, St. Louis, MO, USA), HRP-conjugated anti-rabbit or anti-mouse secondary antibodies (Dianova, Hamburg, Germany), rabbit anti-GFP (ab290, Abcam, Cambridge, UK), sheep anti-mouse and goat anti-rabbit (all Dianova, 1:5000, Hamburg, Germany) coupled to KK114 or Alexa 594 (Atto-Tec, Siegen, Germany), anti-COX2 (ab203912, 1:500, Abcam, Cambridge, UK), anti-ATP5A (ab14748, 1:1000, Abcam, Cambridge, UK), anti-tubulin (ab15246, 1:1000, Abcam, Cambridge, UK), anti-ESR1 (sc-8005, 1:Santa Cruz Biotechnology, Texas, USA).

Validation

All antibodies were validated by us on KO/or knockdown cells.
In addition:
anti-PHB1 validated by Abcam (https://www.abcam.com/en-us/products/primary-antibodies/prohibitin-antibody-ep2803y-ab75766), anti-PHB2 validated by Abcam (https://www.abcam.com/en-us/products/primary-antibodies/rea-antibody-epr14523-ab182139), anti-GFP validated by Clontech (https://www.takarabio.com/products/antibodies-and-elisa/fluorescent-protein-antibodies/green-fluorescent-protein-antibodies), anti-Actin validated by Sigma Aldrich (https://www.sigmaaldrich.com/DE/de/product/sigma/a2228#product-documentation), rabbit anti-GFP validated by Abcam (https://www.abcam.com/en-us/products/primary-antibodies/gfp-antibody-ab290), anti-COX2 validated by Abcam (https://www.abcam.com/en-us/products/primary-antibodies/mtco1-antibody-epr19628-ab203912), anti-ATP5A validated by Abcam (https://www.abcam.com/en-us/products/primary-antibodies/atp5a-antibody-15h4c4-mitochondrial-marker-ab14748), anti-tubulin validated by Abcam (https://www.abcam.com/en-us/products/primary-antibodies/alpha-tubulin-antibody-microtubule-marker-ab15246), anti-ESR1 validated by Santa Cruz Biotechnology (https://www.scbt.com/de/p/estrogen-receptor-alpha-antibody-d-12).

Eukaryotic cell lines

Policy information about cell lines and Sex and Gender in Research

Cell line source(s)

U2OS (Cat. No.: HTB-96, ATCC) Cos-7 (Cat. No.: 87021302, Sigma Aldrich)

Authentication

All by supplier: STR profiling (U2OS), karyotyping (U2OS), morphology (U2OS, Cos-7)

Mycoplasma contamination

negative; cells were tested regularly using extraction-free PCR.

Commonly misidentified lines
(See ICLAC register)

none

Palaeontology and Archaeology

Specimen provenance

Provide provenance information for specimens and describe permits that were obtained for the work (including the name of the issuing authority, the date of issue, and any identifying information). Permits should encompass collection and, where applicable, export.

Specimen deposition

Indicate where the specimens have been deposited to permit free access by other researchers.

Dating methods

If new dates are provided, describe how they were obtained (e.g. collection, storage, sample pretreatment and measurement), where they were obtained (i.e. lab name), the calibration program and the protocol for quality assurance OR state that no new dates are provided.

☐ Tick this box to confirm that the raw and calibrated dates are available in the paper or in Supplementary Information.

Ethics oversight

Identify the organization(s) that approved or provided guidance on the study protocol, OR state that no ethical approval or guidance was required and explain why not.

Note that full information on the approval of the study protocol must also be provided in the manuscript.

Animals and other research organisms

Policy information about studies involving animals; ARRIVE guidelines recommended for reporting animal research, and Sex and Gender in Research

Laboratory animals	Wistar rats
Wild animals	N/A
Reporting on sex	N/A
Field-collected samples	N/A
Ethics oversight	All procedures with live animals were conducted in the animal facility at the Max-Planck-Institute for Multidisciplinary Sciences, Göttingen. According to the German Animal Welfare Law, killing is not an experiment on animals. All requirements of § 4 TierSchG together with § 2 Satz 2, Anlage 1, Abschnitt 2 and Anlage 2 TierSchVersV were implemented.<br><br>The facility is conducted under all aspects of animal welfare. The facility is headed by a veterinarian with special education in laboratory animal science as well as gene technology and molecular genetics.<br>Only professionally educated animal technicians are in charge of animal husbandry and care. The facility is registered according to §11 Abs. 1 TierSchG (Tierschutzgesetz der Bundesrepublik Deutschland, Animal Welfare Law of the Federal Republic of Germany) as documented by 33.23-42508-066-§11, dated Nov 16th, 2023 ("Erlaubnis, zum Halten von Wirbeltieren zur Versuchszwecken", "Permission to keep vertebrates for experimental purposes") by the Niedersächsisches Landesamt für Verbraucherschutz und Lebensmittelsicherheit (Lower Saxony State Office for Consumer Protection and Food Safety). According to the Animal Welfare Law of the Federal Republic of Germany (TierSchG) and the Regulation about animals used in experiments, dated 20th Dec 2022 (TierSchVersV) an animal welfare officer (specialized veterinarian in laboratory animal science) and an animal welfare committee for the institute is established.

Note that full information on the approval of the study protocol must also be provided in the manuscript.

Clinical data

Policy information about clinical studies
All manuscripts should comply with the ICMJE guidelines for publication of clinical research and a completed CONSORT checklist must be included with all submissions.

Clinical trial registration	*Provide the trial registration number from ClinicalTrials.gov or an equivalent agency.*
Study protocol	*Note where the full trial protocol can be accessed OR if not available, explain why.*
Data collection	*Describe the settings and locales of data collection, noting the time periods of recruitment and data collection.*
Outcomes	*Describe how you pre-defined primary and secondary outcome measures and how you assessed these measures.*

Dual use research of concern

Policy information about dual use research of concern

Hazards

Could the accidental, deliberate or reckless misuse of agents or technologies generated in the work, or the application of information presented in the manuscript, pose a threat to:

No	Yes	
☒	☐	Public health
☒	☐	National security
☒	☐	Crops and/or livestock
☒	☐	Ecosystems
☒	☐	Any other significant area

Experiments of concern

Does the work involve any of these experiments of concern:

No	Yes	
☒	☐	Demonstrate how to render a vaccine ineffective
☒	☐	Confer resistance to therapeutically useful antibiotics or antiviral agents
☒	☐	Enhance the virulence of a pathogen or render a nonpathogen virulent
☒	☐	Increase transmissibility of a pathogen
☒	☐	Alter the host range of a pathogen
☒	☐	Enable evasion of diagnostic/detection modalities
☒	☐	Enable the weaponization of a biological agent or toxin
☒	☐	Any other potentially harmful combination of experiments and agents

Plants

Seed stocks	N/A
Novel plant genotypes	N/A
Authentication	N/A

ChIP-seq

Data deposition

☐ Confirm that both raw and final processed data have been deposited in a public database such as GEO.

☐ Confirm that you have deposited or provided access to graph files (e.g. BED files) for the called peaks.

Data access links<br>*May remain private before publication.*	*For "Initial submission" or "Revised version" documents, provide reviewer access links. For your "Final submission" document, provide a link to the deposited data.*
Files in database submission	*Provide a list of all files available in the database submission.*
Genome browser session<br>(e.g. UCSC)	*Provide a link to an anonymized genome browser session for "Initial submission" and "Revised version" documents only, to enable peer review. Write "no longer applicable" for "Final submission" documents.*

Methodology

Replicates	*Describe the experimental replicates, specifying number, type and replicate agreement.*
Sequencing depth	*Describe the sequencing depth for each experiment, providing the total number of reads, uniquely mapped reads, length of reads and whether they were paired- or single-end.*
Antibodies	*Describe the antibodies used for the ChIP-seq experiments; as applicable, provide supplier name, catalog number, clone name, and lot number.*
Peak calling parameters	*Specify the command line program and parameters used for read mapping and peak calling, including the ChIP, control and index files used.*
Data quality	*Describe the methods used to ensure data quality in full detail, including how many peaks are at FDR 5% and above 5-fold enrichment.*
Software	*Describe the software used to collect and analyze the ChIP-seq data. For custom code that has been deposited into a community repository, provide accession details.*

Flow Cytometry

Plots

Confirm that:

☒ The axis labels state the marker and fluorochrome used (e.g. CD4-FITC).

☒ The axis scales are clearly visible. Include numbers along axes only for bottom left plot of group (a 'group' is an analysis of identical markers).

☒ All plots are contour plots with outliers or pseudocolor plots.

☒ A numerical value for number of cells or percentage (with statistics) is provided.

Methodology

Sample preparation	U2OS cells were transfected with the bicistronic nuclease plasmids and the corresponding donor plasmids using FuGENE HD transfection reagent (Promega, Mannheim, Germany). To this end, 20.000 cells per well were seeded in a 6-well plate with supplemented DMEM. The following day, transfection was carried out using a reagent to DNA ratio of 3.5 to 1 and a total DNA amount of 3 µg. Subsequently, the cells were further incubated at 37°C, 5% CO2. After seven days, cells were inspected by fluorescence microscopy. Wells containing cells exhibiting the expected sub-cellular localization of the Dreiklang fusion protein were subjected to single cell sorting into 96-well plates.
Instrument	FACSAria II (BD Biosciences, Heidelberg, Germany)
Software	FACSDiva 6.0 software
Cell population abundance	4.3% for PHB1-Dreiklang knockin cells (Fig. S2E, left); 10% for PHB2-Dreiklang knockin cells (Fig. S2E, right)
Gating strategy	We have identified Dreiklang-expressing knock-in cells by plotting the side scatter area versus fluorescence intensity after excitation with a 488 nm laser (Fig. S2D-E). We have sorted knock-in cells displaying a fluorescence signal that was about 5-fold higher compared to U2OS wildtype cells.

☒ Tick this box to confirm that a figure exemplifying the gating strategy is provided in the Supplementary Information.

Magnetic resonance imaging

Experimental design

Design type	*Indicate task or resting state; event-related or block design.*
Design specifications	*Specify the number of blocks, trials or experimental units per session and/or subject, and specify the length of each trial or block (if trials are blocked) and interval between trials.*
Behavioral performance measures	*State number and/or type of variables recorded (e.g. correct button press, response time) and what statistics were used to establish that the subjects were performing the task as expected (e.g. mean, range, and/or standard deviation across subjects).*

Acquisition

Imaging type(s)	*Specify: functional, structural, diffusion, perfusion.*
Field strength	*Specify in Tesla*
Sequence & imaging parameters	*Specify the pulse sequence type (gradient echo, spin echo, etc.), imaging type (EPI, spiral, etc.), field of view, matrix size, slice thickness, orientation and TE/TR/flip angle.*
Area of acquisition	*State whether a whole brain scan was used OR define the area of acquisition, describing how the region was determined.*

Diffusion MRI ☐ Used ☐ Not used

Preprocessing

Preprocessing software	*Provide detail on software version and revision number and on specific parameters (model/functions, brain extraction, segmentation, smoothing kernel size, etc.).*
Normalization	*If data were normalized/standardized, describe the approach(es): specify linear or non-linear and define image types used for transformation OR indicate that data were not normalized and explain rationale for lack of normalization.*
Normalization template	*Describe the template used for normalization/transformation, specifying subject space or group standardized space (e.g. original Talairach, MNI305, ICBM152) OR indicate that the data were not normalized.*

Noise and artifact removal

Describe your procedure(s) for artifact and structured noise removal, specifying motion parameters, tissue signals and physiological signals (heart rate, respiration).

Volume censoring

Define your software and/or method and criteria for volume censoring, and state the extent of such censoring.

Statistical modeling & inference

Model type and settings

Specify type (mass univariate, multivariate, RSA, predictive, etc.) and describe essential details of the model at the first and second levels (e.g. fixed, random or mixed effects; drift or auto-correlation).

Effect(s) tested

Define precise effect in terms of the task or stimulus conditions instead of psychological concepts and indicate whether ANOVA or factorial designs were used.

Specify type of analysis: ☐ Whole brain ☐ ROI-based ☐ Both

Statistic type for inference

(See Eklund et al. 2016)

Specify voxel-wise or cluster-wise and report all relevant parameters for cluster-wise methods.

Correction

Describe the type of correction and how it is obtained for multiple comparisons (e.g. FWE, FDR, permutation or Monte Carlo).

Models & analysis

n/a	Involved in the study
☐	☐ Functional and/or effective connectivity
☐	☐ Graph analysis
☐	☐ Multivariate modeling or predictive analysis

Functional and/or effective connectivity

Report the measures of dependence used and the model details (e.g. Pearson correlation, partial correlation, mutual information).

Graph analysis

Report the dependent variable and connectivity measure, specifying weighted graph or binarized graph, subject- or group-level, and the global and/or node summaries used (e.g. clustering coefficient, efficiency, etc.).

Multivariate modeling and predictive analysis

Specify independent variables, features extraction and dimension reduction, model, training and evaluation metrics.
