# Supplementary figures and images for: In situ architecture of the human prohibitin complex
Source: Nat Cell Biol. 2025 Mar 21;27(4):633–40. doi: 10.1038/s41556-025-01620-1 (PMC11991916; doi:10.1038/s41556-025-01620-1)

**Figure S3, Panel A and B**

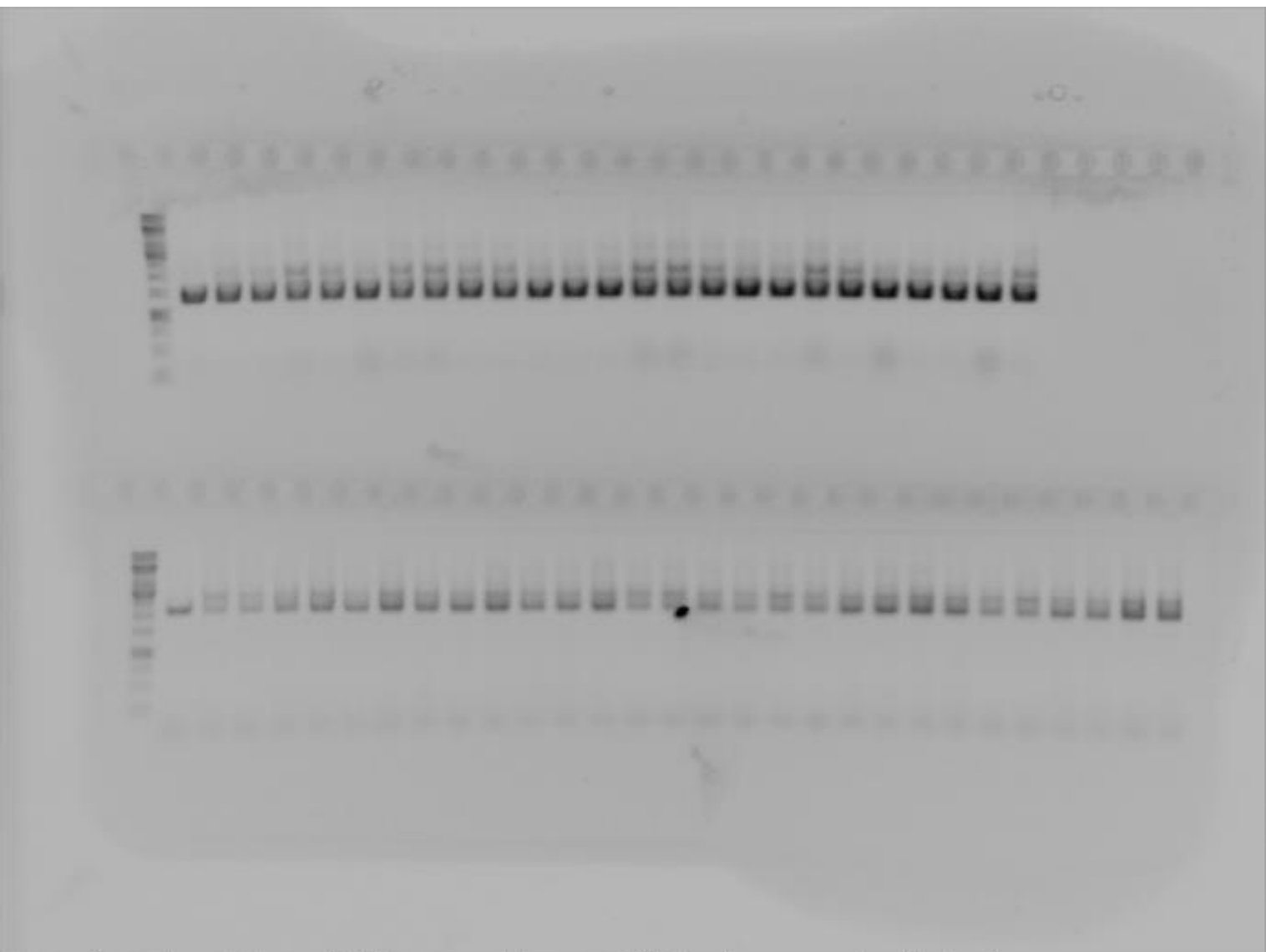

User1 Exp. Time: 0.04 sec Upper: 255 Lower: 0 Int.: 0  
Date: 12.03.2016 Time: 20:54:54

Figure S3, Panel C and D

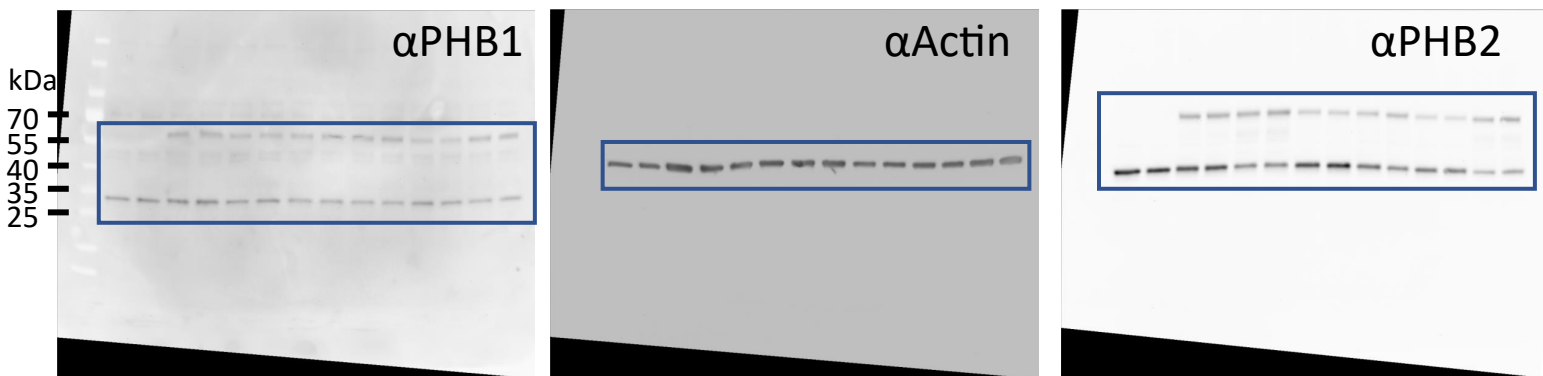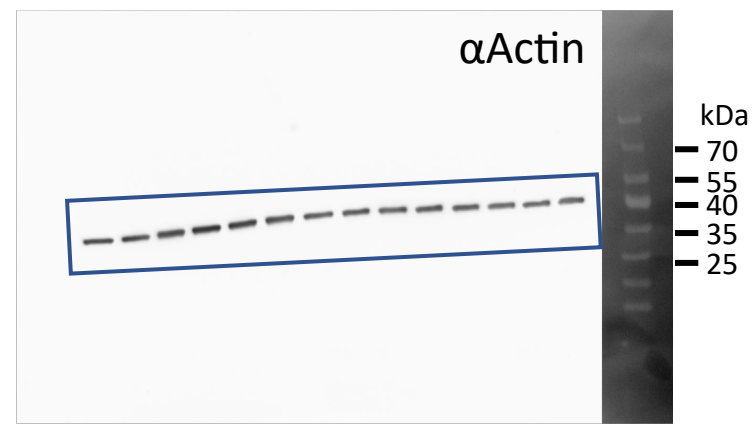

Supplement: Supplementary file 8 — Unprocessed blots. [file 41556_2025_1620_MOESM8_ESM.pdf]

**Figure S6, Panel B**

$\alpha$ PHB1

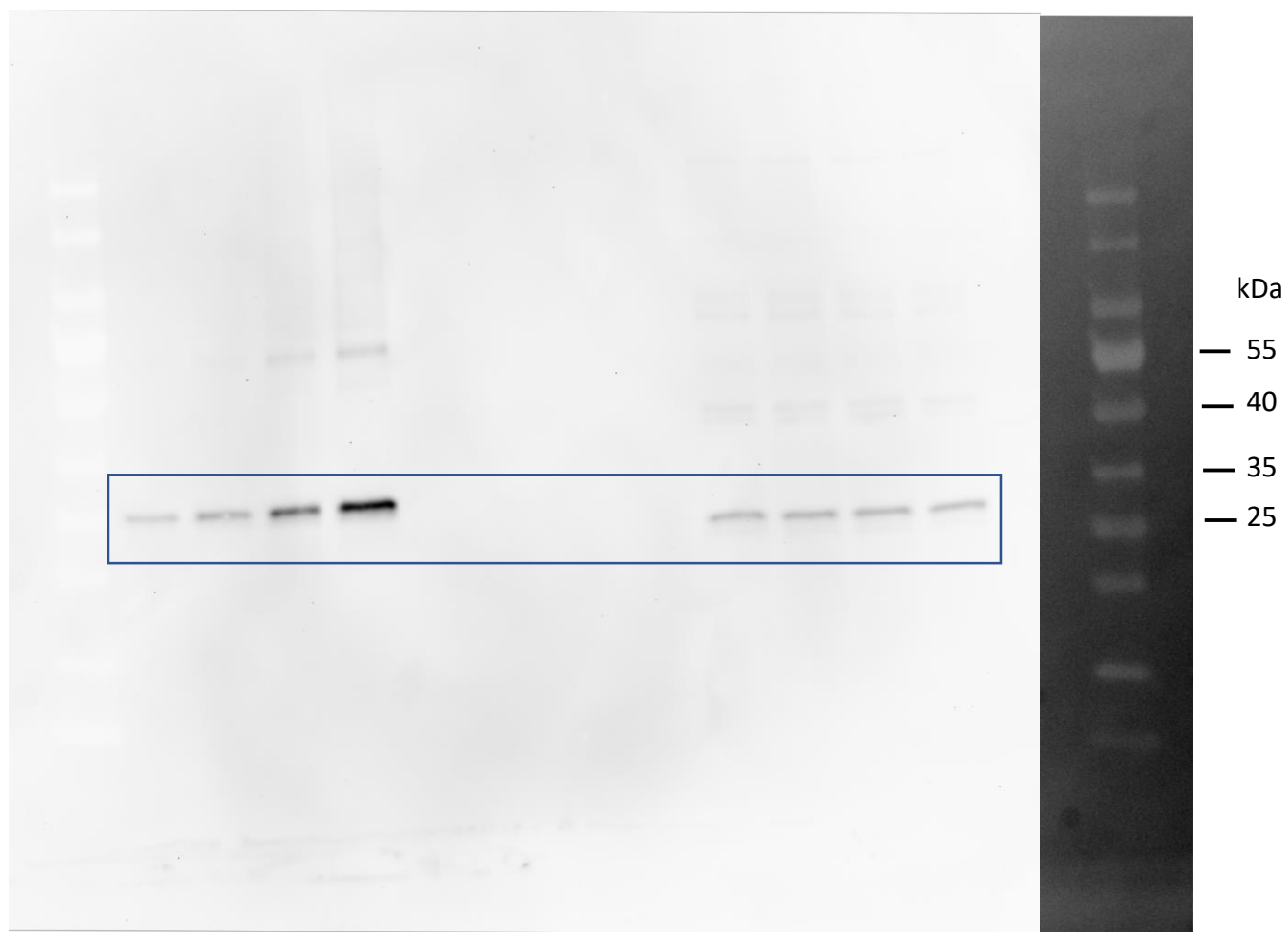

$\alpha$ PHB2

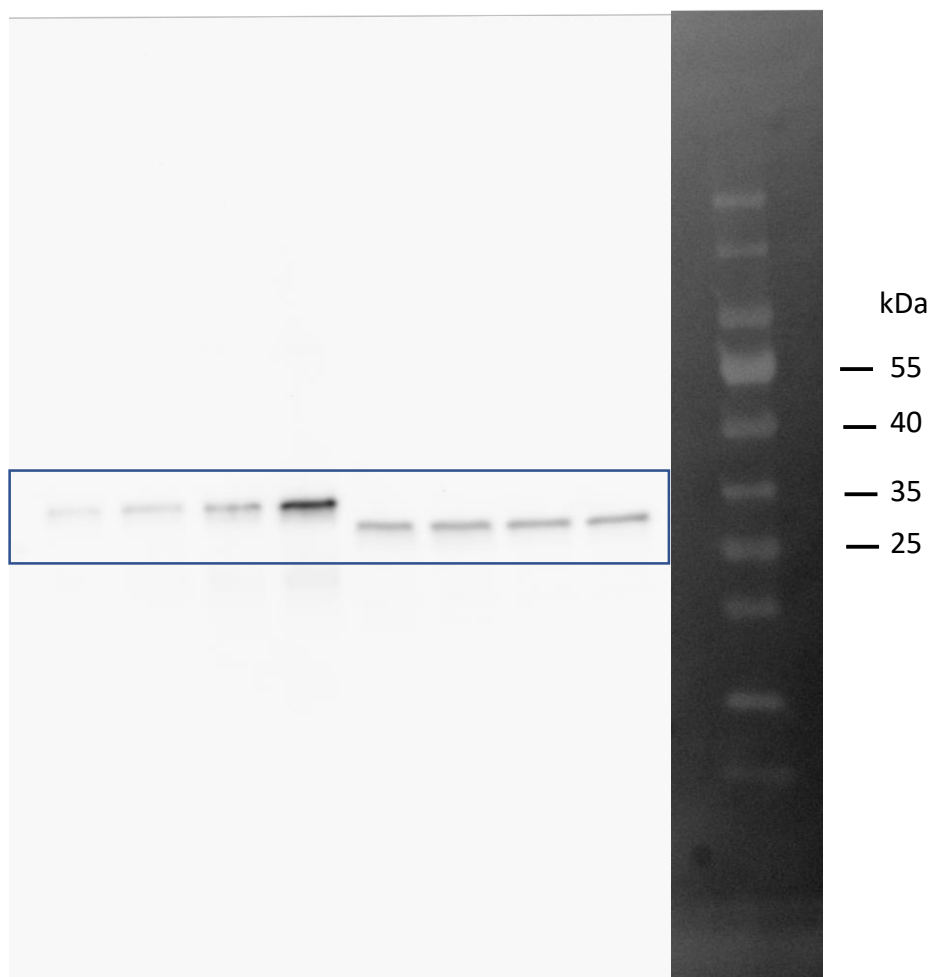

Supplement: Supplementary file 11 — Unprocessed blots. [file 41556_2025_1620_MOESM11_ESM.pdf]
