# Supplementary material for: In situ architecture of the human prohibitin complex
Source: Nat Cell Biol. 2025 Mar 21;27(4):633–40. doi: 10.1038/s41556-025-01620-1 (PMC11991916; doi:10.1038/s41556-025-01620-1)

Figure S5, Panel A and B

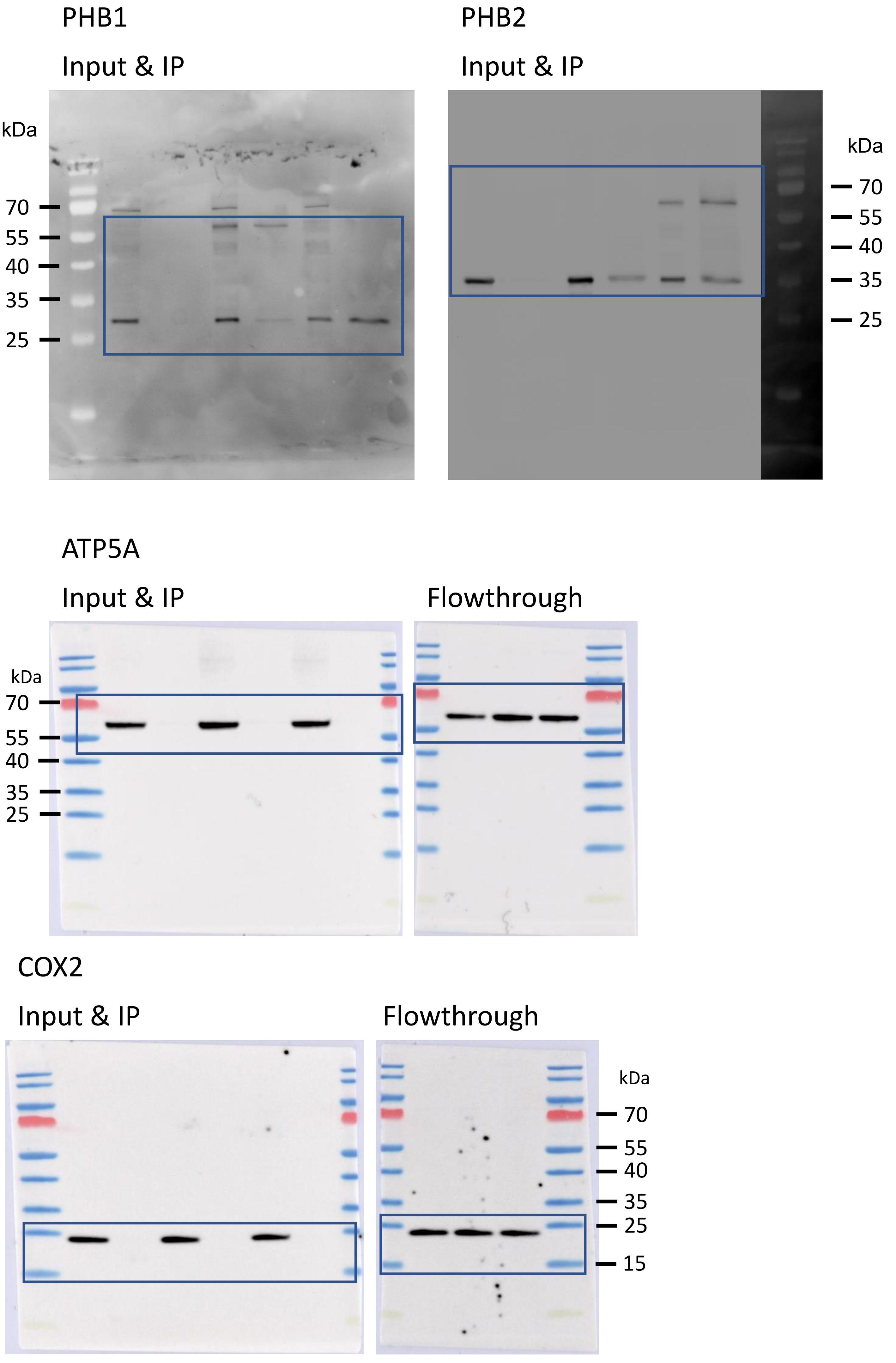

Supplement: Supplementary file 9 — Unprocessed blots. [file 41556_2025_1620_MOESM9_ESM.pdf]
