# Supplementary material for: In situ architecture of the human prohibitin complex
Source: Nat Cell Biol. 2025 Mar 21;27(4):633–40. doi: 10.1038/s41556-025-01620-1 (PMC11991916; doi:10.1038/s41556-025-01620-1)

Figure S7, Panel B

PHB knockdown

αTubulin

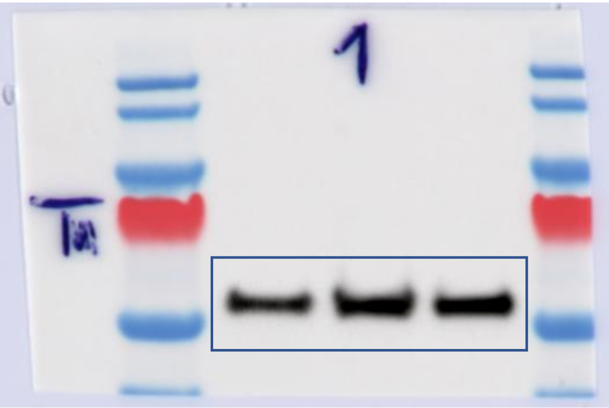

αTubulin

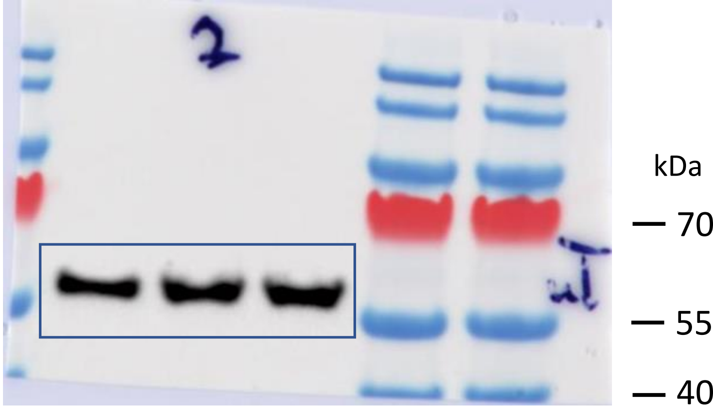

αPHB1

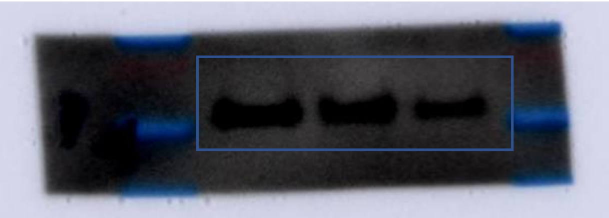

αPHB2

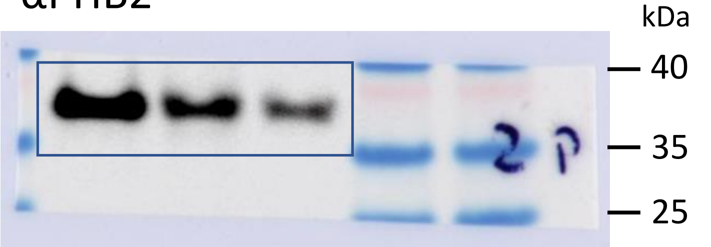

αPHB1 (greyscale)

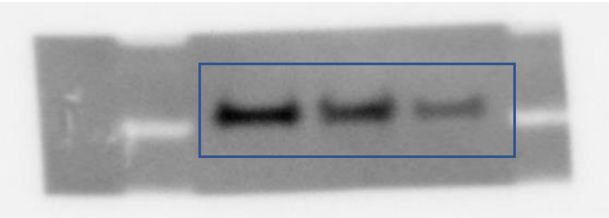

Supplement: Supplementary file 13 — Unprocessed blots. [file 41556_2025_1620_MOESM13_ESM.pdf]
